# Epuisement du capital vasculaire en hémodialyse: quelle issue?

**DOI:** 10.11604/pamj.2016.25.237.10665

**Published:** 2016-12-19

**Authors:** Meriam Hajji, Amel Harzallah, Hayet Kaaroud, Mona Jerbi, Soumaya Chargui, Fethi El Younsi, Fethi Ben Hamida, Taieb Ben abdallah

**Affiliations:** 1 Service de Médecine Interne A, Hôpital Charles Nicolle, Tunis, Tunisie; 2 Laboratoire de Pathologie Rénale LR00SP01, Hôpital Charles Nicolle, Tunis, Tunisie; 3 Faculté de Médecine de Tunis, université Tunis El Manar, Tunis, Tunisie

**Keywords:** Hémodialyse, accès vasculaire, thrombose, Hemodialysis, vascular access, thrombosis

## Abstract

Malgré les progrès réalisés dans le traitement de l’insuffisance rénale chronique, l’accès vasculaire reste le maillon faible dans la thérapie de suppléance extrarénale et la principale source de morbidité chez les patients hémodialysés. Nous rapportons l’observation d’une jeune patiente ayant une insuffisance rénale chronique secondaire à une néphropathie vasculaire en hémodialyse périodique, confrontée précocement à un épuisement de son capital vasculaire, en raison de thromboses itératives des fistules artério-veineuses et l’échec de la dialyse péritonéale. Un déficit en protéine C a été objectivé. Elle a bénéficié de la mise en place d’un cathéter tunnelisé au niveau de l’oreillette droite par thoracotomie antéro-latérale droite à travers la veine cave inférieure non fonctionnel au bout de trois mois de son utilisation. Elle est depuis dialysée par ponction des veines jugulaires externes.

## Abstract (en)

Despite advances in the treatment of chronic renal failure, vascular access remains the weakest link in renal replacement therapy (RRT) and the leading cause of morbidity in patients on hemodialysis We report the case of a young female patient with chronic renal insufficiency secondary to vascular nephropathy on periodic hemodialysis and whose vascular capital was early exhausted due to iterative thromboses in arteriovenous fistulas and failure in peritoneal dialysis. Protein C deficiency was objectified. The patient underwent tunneled hemodialysis catheter insertion at the level of the right atrium via a right anterolateral thoracotomy with cannulation of the inferior vena cava, with poor functional outcome after three months of use. Since then she has been dialyzed using puncture of the external jugular veins.

## Introduction

Les fistules artério-veineuses constituent l’accès vasculaire de référence et de première intention en dialyse. Les pontages artério-veineux et les cathéters veineux tunnelisés sont utilisés en cas d’épuisement des sites anatomiques par des accès vasculaires antérieurs multiples. Nous rapportons l’observation d’une patiente présentant un épuisement de son capital vasculaire.

## Patient et observation

Il s’agit de Mme J.S âgée de 40 ans, sans antécédents, ayant présenté à l’âge de 24 ans une grossesse compliquée d’une toxémie gravidique avec un hématome rétro placentaire et une mort fœtale in utéro occasionnant une insuffisance rénale aigue. L’histologie rénale a conclu à une nécrose corticale. L’évolution était marquée par la dégradation progressive de sa fonction rénale. Le stade terminal de l’insuffisance rénale était atteint au bout de 6 ans. Elle était initialement hémodialysée via une fistule artério-veineuse (FAV) huméro-basilique droite pendant 6 mois vu la qualité des vaissaux grêle à la phlébographie, puis par l’intermédiaire d’une fistule huméro-céphalique gauche pendant une année. Plusieurs fistules ont par la suite été réalisées, suite à la thrombose itérative de ces accès vasculaires. Entre-temps, il n’a pas eu de tentatives de thrombectomies. Le bilan de thrombophilie, comportant le dosage du taux de Protéine C et S, antithrombine III, homocystéine, ainsi que la recherche des anticorps anti Beta 2 glycoprotein 1 et des anticorps anti-phospholipides, réalisé initialement était négatif. Le switch en dialyse péritonéale a été préconisé au bout de cinq ans marqué par la survenue ultérieurement de plusieurs épisodes de péritonites. La cartographie artérielle et veineuse a révélé une thrombose du tronc inominé droit avec une importante circulation veineuse collatérale cervicale, une infiltration athéromateuse marquée des artères humérales, radiales et cubitales, une thrombose veineuse étendue et bilatérale des veines fémorales communes et superficielle ainsi qu’une thrombose des deux artères fémorales superficielles dans leur tiers moyen. La confection d’une FAV au niveau des membres inférieurs était jugée impossible. Une transplantation rénale n’était pas envisageable également vu la multitude des thromboses et la qualité du réseau veineux abdomino-pelvien.

La dialyse a été par la suite assurée par la ponction des deux veines jugulaires externes pendant six mois. Elle était mise également sous traitement anti-aggrégant, qui a été arrêté vu le saignement important et le temps de compression allongé au retrait des aiguilles. Elle a bénéficié ultérieurement de la mise en place d’un pontage prothétique (Gortex) axillo-axillaire gauche qui s’est thrombosé au bout de deux ans. La dialyse péritonéale a été de nouveau indiquée, mais le cathéter (KT) était déplacé. Le repositionnement du KT s’est compliqué par la survenue d’une septicémie dont le germe n’a pu être identifié. L’évolution était favorable après ablation du KT et traitement antibiotique.

**Figure 1:**
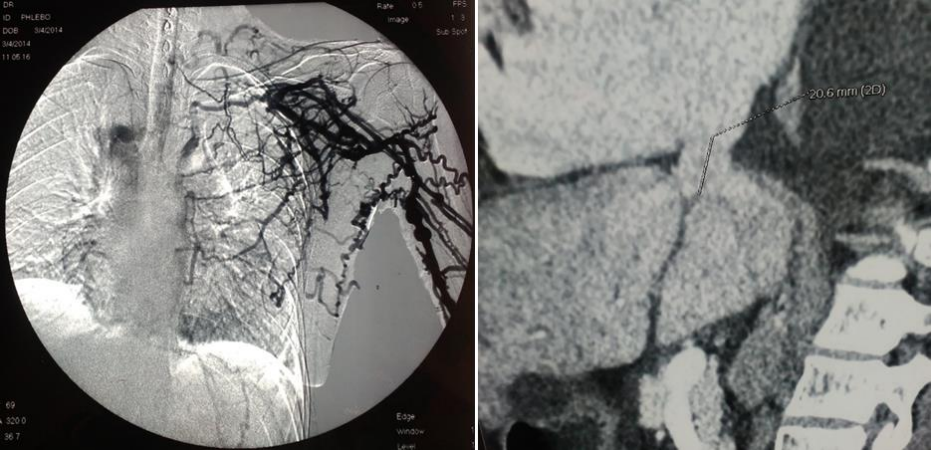
Thromboses veineuses du membre supérieur

**Figure 2:**
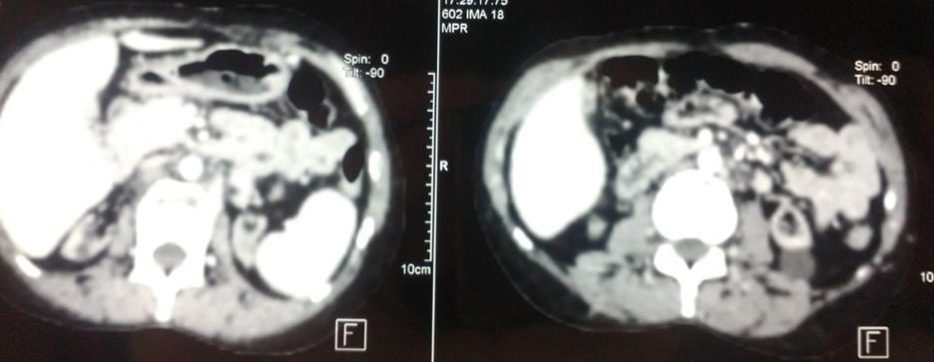
Thrombose de la veine cave supérieure

Une nouvelle cartographie a été réalisée, montrant une thrombose complète de la veine sous-clavière droite et partielle de la veine sous clavière gauche (Figure 1), ainsi qu’une thrombose des deux veines fémorales, des veines iliaques primitives, et de la veine cave supérieure (Figure 2). La mise en place d’une prothèse type PTFE ilio-rénale a été indiquée mais récusée en per-opératoire devant la présence d’une importante hépatomégalie avec saignement en nappe et des varicosités en rapport avec une hypertension portale. On a continué à dialyser la patiente par double ponction des veines jugulaires externes. Elle a bénéficié par la suite de la mise en place d’un cathéter tunnelisé au niveau de l’oreillette droite, par thoracotomie antéro-latérale droite à travers la veine cave inférieure. Les suites opératoires étaient simples. Au bout de trois mois, le KT n’était plus fonctionnel en raison d’une thrombose à son niveau. Un deuxième bilan de thrombophilie a été réalisé révélant un déficit en protéine C (42%). Ainsi, la patiente était mise sous traitement anticoagulant. L’angio-scanner thoraco-abdominal a mis en évidence une veine cave inférieure grêle avec la présence d’une sténose régulière à son niveau. Aucune alternative thérapeutique n’a pu être proposée par les chirurgiens cardio-vasculaires, et la patiente n’acceptait plus aucune intervention vasculaire. La dialyse est assurée depuis par la ponction des deux veines jugulaires externes. Après un recul de 18 mois, elle se porte relativement bien.

## Discussion

Notre observation illustre les difficultés de l’accès vasculaire depuis l’amélioration de la survie en dialyse et en l’absence d’une transplantation rénale. Les complications de l’accès vasculaire en hémodialyse sont fréquentes et représentent la première cause d’hospitalisation [1]. Notre patiente a présenté plusieurs thromboses veineuses au niveau des deux membres supérieurs, puis on a eu recours aux prothèses vasculaires et aux cathéters veineux. En effet, Il est recommandé de recourir d´abord à des fistules natives avant les prothèses vasculaires [2, 3]. Les complications des FAV pour hémodialyse chronique constituent la principale cause de morbidité chez 1´hémodialysé chronique [4]. Il est primordial de s´appliquer lors de leur création, et d´accorder le maximum d´attention lors de leur manipulation. Ceci implique le chirurgien, le néphrologue, les infirmiers et le patient lui-même, ainsi que la mise en place d´un programme de surveillance des FAV. Les techniques d´angioplastie endoluminale offrent les meilleures perspectives dans la gestion des complications [5]. Le problème de thromboses itératives de FAV posé par notre patiente, pourrait être prévenue par une surveillance régulière de la voie d´abord au cours des dialyses et par la pratique rapproché d’un doppler veineux afin de diagnostiquer à temps la thrombose et de traiter à temps la FAV. En effet, le traitement utilise principalement les techniques endovasculaires en première intention [6]. Le traitement agressif de toutes les complications de FAV est capital dans la survie de ces malades. Néanmoins, notre patiente n’a pas bénéficié ni de surveillance rigoureuse clinique et échographique de ses FAV, ni de traitement visant à sauver la FAV confectionnée. On s’est contenté d’une succession de passage à d´autres types d´accès vasculaires. Il convient de souligner l’intérêt majeur du traitement médical pour prévenir et traiter les thromboses de FAV. En effet, L’aspirine semble montrer un intérêt dans la prévention de la thrombose de FAV, avec une diminution du risque de thrombose [7]. L’étude de Dixon et al retrouve un bénéfice primaire à l’association aspirine et dipyridamol avec une augmentation de la durée de vie de l’abord avant première réintervention, mais il n’y avait pas de différence de survie totale de l’abord. Le nombre d’évènements indésirables dans les deux groupes n’était pas différent [8]. Le clopidogrel quant a lui a montré un intérêt dans la prévention de thrombose de pontages prothétiques, tout en améliorant leur durée totale d’utilisation [9]. L’étude de Dember et al retrouve un intérêt au clopidogrel dans la prévention de thromboses des FAV, mais il n’existe pas de bénéfice au long cours, avec une survie d’abord veineux comparable [10]. L’étude de Kaufman et al. ne montre pas de bénéfice supplémentaire à l’utilisation du clopidogrel en plus de l’aspirine chez les patients porteur de FAV dans la prévention thrombotique [11]. Cependant, l’aspirine et le clopidogrel n’ont pas l’AMM dans les préventions primaires ou secondaires de thrombose d’abord. Ils sont pourtant parfois utilisés en pratique pour ces indications.

L’épuisement du capital vasculaire n’est pas rare pour les patients les plus anciens en dialyse [12]. Notre jeune patiente était hémodialysée depuis plus que 5 ans. De plus, elle avait une anomalie du bilan de thrombophilie prédisposant au risque de thromboses itératives. Il s’agissait d’un trouble héréditaire de la coagulation associé à un risque accru de thromboses veineuses en raison d´une synthèse réduite et/ou d´une baisse d´activité de la protéine C [13]. Il convient ainsi, de rechercher systématiquement un état d’hypercoaguabilité, lorsque les thromboses sont répétées ou se produisant en dehors d’une sténose patente.

L’hémodialyse par cathétérisme Intra-auriculaire a été rapportée dans la littérature, elle constitue une mesure de sauvetage sûre et efficace pour les patients atteints d´insuffisance veineuse et sans aucune possibilité de dialyse péritonéale ou de transplantation rénale [14, 15]. Cette alternative a ainsi été réalisée chez notre patiente devant l’échec de la dialyse péritonéale vu les multiples complications infectieuses et l’impossibilité d’une transplantation rénale devant la qualité médiocre du réseau veineux abomino-pelvien. La thrombose de ce type de cathéter a été rapportée dans trois cas (11,1%) dans la littérature [16]. Dans d’autres études, l’oreillette droite a constitué une voie plus privilégiée que la veine cave supérieure pour l’emplacement du cathéter de dialyse tunnelisé [17, 18]. Cependant, les recommandations de pratique clinique ne sont pas tous unanimes pour le choix de cet accès vasculaire [17, 19].

## Conclusion

Le nombre de patients hémodialysés ne cesse d'augmenter chaque année. L’abord vasculaire pour la dialyse est au centre de la prise en charge globale du patient. L’épuisement du capital vasculaire notamment au jeune âge, constitue un véritable challenge thérapeutique tant pour le néphrologue que pour le chirurgien cardio-vasculaire. La transplantation rénale demeure la solution idéale pour la préservation du capital veineux.
